# Quantification of the size of subchorionic hematoma causing pregnancy-related complications: a retrospective cohort study

**DOI:** 10.1007/s10396-024-01488-y

**Published:** 2024-08-27

**Authors:** Tatsuya Yoshihara, Yasuhiko Okuda, Osamu Yoshino

**Affiliations:** Department of Obstetrics and Gynecology, Faculty of Medicine, University of Yamanashi, Yamanashi, Japan

**Keywords:** First trimester, Pregnancy outcome, Preterm delivery, Subchorionic hematoma, Subchorionic hemorrhage

## Abstract

**Purpose:**

This study aimed to establish criteria for defining “large” subchorionic hematoma (SCH) and assess its association with pregnancy complications.

**Method:**

This was a retrospective cohort study conducted at our institution between 2019 and 2020. We compared the size of SCH between the pregnancy-related complication and non-complication groups, using two measurement methods. Receiver operating characteristic (ROC) curve analysis determined cutoff values. Additionally, we compared the occurrence of pregnancy complications among three groups: large SCH group (above the cutoff value), non-large SCH group (below the cutoff value), and non-SCH group.

**Results:**

Of 1305 singleton pregnancies managed during the study, 80 cases were diagnosed with SCH. Pregnancy complications occurred in 15 patients. The patients with pregnancy complications had significantly larger SCH sizes with both measurement methods. For each method, the cutoff values calculated from the ROC curve analysis were as follows: Method 1, 25% (area under the ROC curve [AUC], 0.662); Method 2, 30% (AUC, 0.624). In Method 1, we found a significantly higher occurrence of preterm delivery in the large SCH group (24.1%) than in the non-large SCH (4.2%) and non-SCH groups (5.3%; all p < 0.01). In Method 2, there was a significantly higher occurrence of preterm delivery in the large SCH group (33.3%) than in the non-large SCH (6.5%) and non-SCH groups (5.3%; all p < 0.01).

**Conclusion:**

Large SCHs may indicate a high risk of pregnancy-related complications. Among these, recognizing and managing cases that exceed the aforementioned cutoff value as high-risk cases may be beneficial for reducing pregnancy complications.

**Supplementary Information:**

The online version contains supplementary material available at 10.1007/s10396-024-01488-y.

## Introduction

Subchorionic hematoma (SCH) is a complication of pregnancy that occurs between the first and second trimesters. However, the definitions and diagnoses are uncertain. Therefore, the incidence reported in previous studies varies (0.5–40% of all pregnancies) [1–3]. Clinically, SCH is often diagnosed when hypoechoic areas are observed in addition to the amniotic cavity during transabdominal or transvaginal ultrasonography [4]. The cause of SCH remains unclear. However, it is thought that during placentation, when the villi invade the desmoplasia, they injure blood vessels and form a hematoma [5].

It is a commonly encountered complication that resolves spontaneously and has no effect on pregnancy outcomes in many cases. [3] However, compared to cases without SCH, pregnancy complications are reportedly frequent, such as miscarriage, preterm delivery, preterm premature rupture of the membrane (PROM), fetal growth restriction (FGR), and placental abruption [6, 7]. Risk factors for these pregnancy complications are SCH not resolving until the second trimester, complications of chorioamnionitis, and large hematoma size [8]. Persistence of the hematoma can be assessed with ultrasonography, and there are diagnostic and assessment methods for chorioamnionitis, such as the clinical chorioamnionitis criteria proposed by Lencki et al., [9] and Triple I, proposed by a National Institute of Child Health and Human Development expert panel to replace the term chorioamnionitis [10]. However, what exactly constitutes a “large” hemorrhage is debatable, and there is ambiguity regarding the specific extent that qualifies as “large.”

**Fig. 1:**
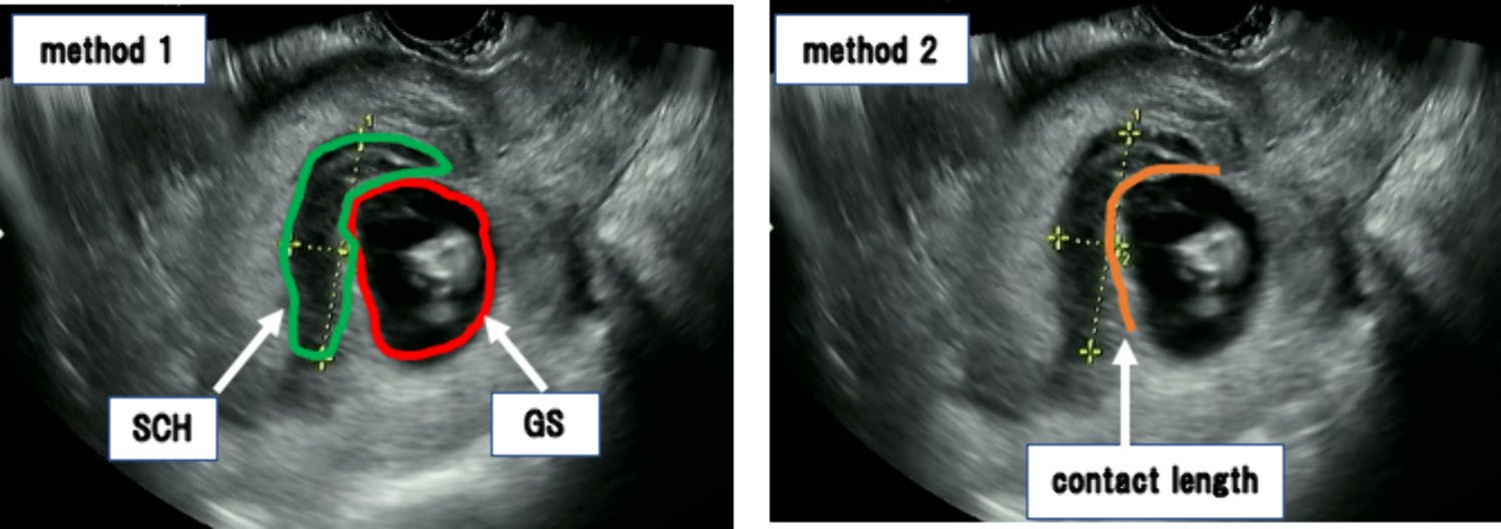
SCH was quantified using two different methods. In Method 1, we measured SCH size in relation to the size of the GS or amniotic cavity area. In Method 2, we measured the SCH contact length in relation to the GS or amniotic cavity circumference. An SCH is defined as a hypoechoic area distinct from the amniotic cavity. *GS* gestational sac, *SCH* subchorionic hematoma

SCH size has been evaluated using various methods such as SCH size relative to the gestational sac (GS) size (Fig. 1, Method 1) [3, 8, 11–14], SCH contact length relative to the GS circumference (Fig. 1, Method 2) [4, 8, 11, 12], and calculation of SCH volume from ultrasound measurements [15, 16]. However, no reports have indicated the threshold beyond which SCH size is related to a high risk of developing pregnancy complications. Furthermore, there has been no exploration of the most suitable method for predicting pregnancy complications.

Here, we quantified the size of hematomas using ultrasonography and identified a high-risk group based on the size at which SCHs pose a risk for pregnancy-related complications. We also compared the incidence of pregnancy-related complications between the SCH and non-SCH groups.

## Methods

This retrospective cohort study was conducted at the University of Yamanashi Hospital between January 2019 and December 2020, and was approved by the Ethics Committee of the University of Yamanashi (approval number: R 2776). The opportunity to provide information about this research and to determine the consent for questions and information usage has been provided through the opt-out option.

This study targeted singleton pregnancies managed at our institution, specifically those diagnosed with SCH during the first trimester. SCH was diagnosed using transvaginal or transabdominal ultrasonography (Voluson P8 and E10; GE HealthCare, Chicago, IL, USA). SCH was defined as a hypoechoic area distinct from the amniotic cavity, which the attending physician had diagnosed as a hematoma. Vaginal bleeding was not considered during the diagnosis. From the ultrasound findings, it was confirmed that the hypoechoic area was not a mole. The group categorized as having pregnancy-related complications included patients with SCH who experienced miscarriage, preterm delivery, preterm PROM, FGR, or placental abruption. FGR was diagnosed as ‘small for gestational age’ based on measurements obtained after birth. Placental abruption was diagnosed by an attending physician during delivery.

In Examination 1, we compared the size of the SCH between the pregnancy-related complication and non-complication groups. SCH size was quantified using two established methods based on previous reports, utilizing the ultrasound image where the SCH appeared largest (Fig. 1) [3, 4, 8, 11–14]. In Method 1, we measured SCH size in relation to the size of the GS or amniotic cavity area. In Method 2, we measured the SCH contact length in relation to the GS or amniotic cavity circumference. In this study, the measurements were performed by a single examiner (T.Y.) using imaging data from cases diagnosed with SCH. When SCH was detected over multiple periods, the period during which SCH showed the maximum value in both Method 1 and Method 2 was adopted. Additionally, we employed receiver operating characteristic (ROC) curve analysis to calculate the cutoff value for the occurrence of pregnancy complications.

In Examination 2, we compared the incidence of pregnancy complications between the SCH and non-SCH groups. The non-SCH group consisted of patients managed at our institution in 2019. The maternal background (age, parity, and use of assisted reproductive technology [ART]) of patients in the SCH group was matched using propensity score matching. Furthermore, we compared the occurrence of pregnancy complications among three groups: the group that exceeded the cutoff value calculated in Examination 1 (large SCH group), the group with values below the cutoff value (non-large SCH group), and the non-SCH group.

### Statistical analysis

JMP Pro (version 17.2.0) was used for statistical analyses. Data for continuous variables are presented as mean ± standard deviation. The chi-squared test, Mann–Whitney U test, and Fisher’s exact test were used for analysis, with p < 0.05 indicating a significant difference. Comparisons among the three groups were conducted using the Kruskal–Wallis rank sum test. For items showing significance, a post hoc test using Steel–Dwass multiple comparisons was employed to ascertain the source of the differences. Additionally, receiver operating characteristic (ROC) curve analysis was performed to calculate the cutoff value for the occurrence of pregnancy complications.

## Results

During the research period, we managed the pregnancies and deliveries of 1,305 singleton pregnancies. Of those, 80 (6.1%) patients were diagnosed with SCH in the first trimester. The mean age of patients diagnosed with SCH was 34.4 ± 4.1 years. Thirty-seven (46.3%) of the 80 patients underwent ART.

Pregnancy complications occurred in 15 (18.9%) patients. The patients, including duplicates, consisted of three, nine, three, three, and zero cases of miscarriage, preterm delivery, preterm PROM, FGR, and placental abruption, respectively (Supplement Table 1).

**Table 1:** Comparison of SCH size between the group with pregnancy-related complications and that without complications

	Complications (n = 15)	Non-complications (n = 65)	p-value
Method 1	80.7 ± 131.3%	32.1 ± 52.8%	0.02^*^
Method 2	28.2 ± 19.8%	19.0 ± 11.8%	0.02^*^

Pregnancy-related complications (n = 15) and non-complications (n = 65) are shown. Data are presented as mean ± SD and ratios

*SCH* subchorionic hematoma, *SD* standard deviation

Moreover, p-values were assessed using the Mann–Whitney *U* test (*p < 0.05)

**Fig. 2:**
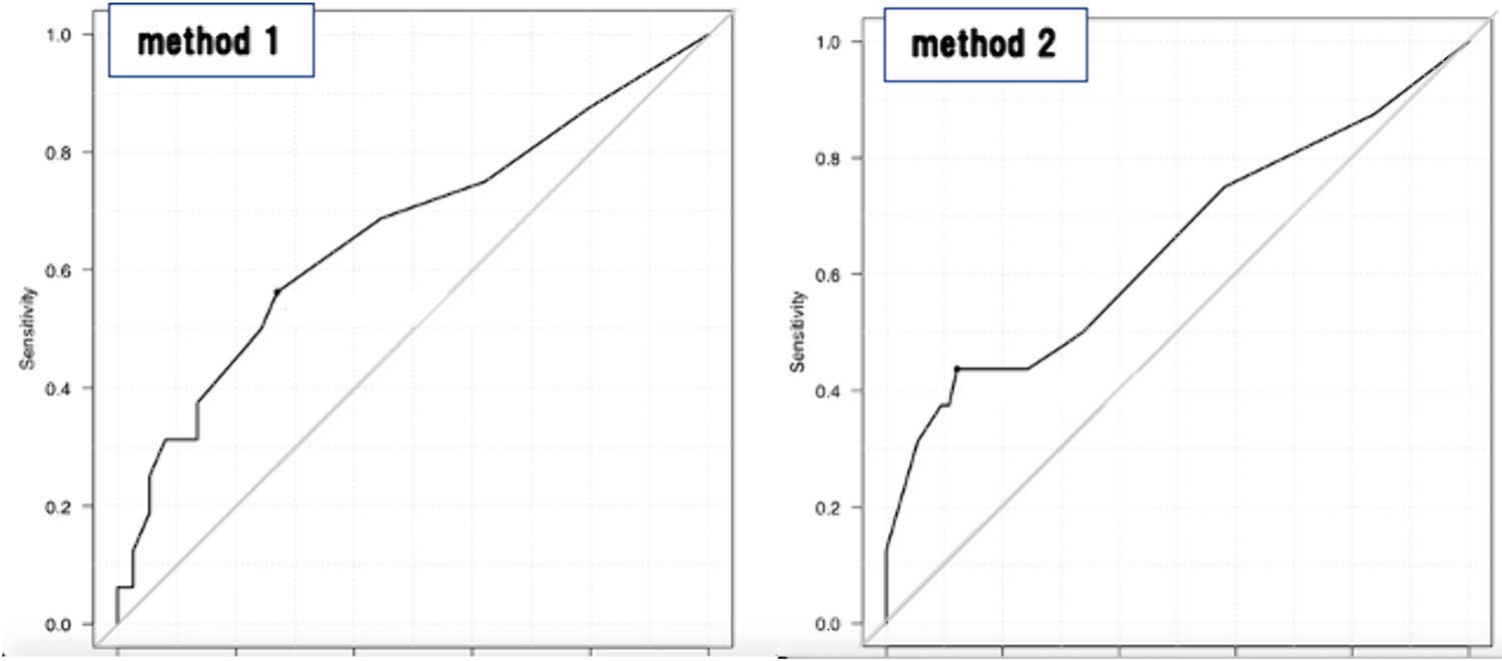
The ROC curves for each method. ROC curves by SCH sizing method. Methods 1 and 2 are depicted in the left and right panels, respectively. *ROC* receiver operating characteristic, *SCH* subchorionic hematoma

Examination 1 revealed no differences between the pregnancy-related complication and non-complication groups in terms of maternal background, history of previous preterm delivery, and pre-pregnancy body mass index (Supplement Table 2). Those with pregnancy-related complications had significantly larger SCH sizes with both measurement methods (Method 1: 80.7 ± 131.3% vs. 32.1 ± 52.8%, p = 0.02; Method 2: 28.2 ± 19.8% vs. 19.0 ± 11.8%, p = 0.02) (Table 1). For each method, the cutoff values calculated from the ROC analysis were as follows: Method 1, 25% (sensitivity, 0.64; specificity, 0.67; area under the ROC curve [AUC], 0.662); and Method 2, 30% (sensitivity, 0.43; specificity, 0.84; AUC, 0.624) (Fig. 2).

**Table 2:** Comparison of gestational age at delivery and the occurrence of pregnancy-related complications between SCH and non-SCH cases

	SCH (n = 77)	Non-SCH (n = 77)	p-value
GA of delivery	38.3 ± 2.4	38.7 ± 2.1	0.31
Preterm delivery	11.3%	5.3%	0.25
Preterm PROM	3.8%	1.3%	0.37
FGR	5.0%	6.6%	0.74
Placental abruption	0	1.5%	0.61

Patients with SCH (n = 77) and without SCH (n = 77) are shown. Patients with miscarriages were excluded from the analysis. The non-SCH group consisted of patients managed at our institution in 2019. Maternal background (age, parity, and use of ART) was matched with the SCH group using propensity score matching. Data are presented as mean ± SD and ratios

*SCH* subchorionic hematoma, *ART* assisted reproductive technology, *SD* standard deviation, *GA* gestational age, *PROM* premature rupture of membranes, *FGR* fetal growth restriction

Moreover, p-values were assessed using the chi-square test and Mann–Whitney *U* test (*p < 0.05)

In Examination 2, comparisons between the SCH and non-SCH groups were as follows: preterm delivery (SCH group 11.3% vs. non-SCH group 5.3%, p = 0.25), preterm PROM (SCH group 3.8% vs. non-SCH group 1.3%, p = 0.37), FGR (SCH group 5.0% vs. non-SCH group 6.6%, p = 0.74), and placental abruption (SCH group 0% vs. non-SCH group 1.5%, p = 0.61). No significant differences were observed for any of the variables (Table 2).

In Method 1, 29 of 77 patients (37.6%) exceeded the cutoff value. When comparing the large, non-large, and non-SCH groups, a significant association with preterm delivery was observed (p < 0.01; Table 3). When conducting a post-hoc test to identify the source of the differences, we found a significantly higher occurrence of preterm delivery in the large SCH group (seven cases, 24.1%) as compared with the non-large SCH (two cases, 4.2%) and non-SCH groups (four cases, 5.3%; all p < 0.01). Early preterm delivery before 34 weeks of gestational age occurred in three cases in the large SCH group, no cases in the non-large SCH group, and two cases in the non-SCH group.

**Table 3:** Comparison of gestational age at delivery and the occurrence of pregnancy-related complications between the cases of SCH that exceeded the cutoff value in Method 1 and non-SCH cases

	Large SCH (n = 29)	Non-large SCH (n = 48)	Non-SCH (n = 77)	p-value
GA of delivery	37.5 ± 3.4	38.8 ± 1.4	38.7 ± 2.1	0.24
Preterm delivery	24.1%	4.2%	5.3%	< 0.01^*^
Preterm PROM	3.4%	4.2%	1.3%	0.59
FGR	6.9%	4.2%	6.6%	0.81
Placental abruption	0	0	1.3%	0.61

Patients with SCH that exceeded the cutoff value (n = 29), cases of SCH below the cutoff value (n = 48), and non-SCH cases (n = 77) are shown. Patients with miscarriages were excluded from the analysis. Data are presented as mean ± SD and the ratio

*SCH* subchorionic hematoma, *SD* standard deviation, *GA* gestational age, *PROM* premature rupture of membranes, *FGR* fetal growth restriction

Moreover, p-values were assessed using the Kruskal–Wallis rank-sum test (*p < 0.05)

In Method 2, among the 77 cases, 15 (19.5%) exceeded the cutoff value. When comparing the large, non-large, and non-SCH groups, a significant association with preterm delivery was observed (p < 0.01; Table 4). When conducting a post hoc test to identify the source of differences, we found a significantly higher occurrence of preterm delivery in the large SCH group (five cases, 33.3%) as compared with the non-large SCH (four cases, 6.5%) and non-SCH groups (four cases, 5.3%; p < 0.01). Early preterm delivery before 34 weeks of gestational age occurred in two cases in the large SCH group, one case in the non-large SCH group, and two cases in the non-SCH group.

**Table 4:** Comparison of gestational age at delivery and the occurrence of pregnancy-related complications between the cases of SCH that exceeded the cutoff value in Method 2 and non-SCH cases

	Large SCH (n = 15)	Non-large SCH (n = 62)	Non-SCH (n = 77)	p-value
GA of delivery	36.6 ± 3.9	38.8 ± 1.7	38.7 ± 2.1	0.06
Preterm delivery	33.3%	6.5%	5.3%	< 0.01^*^
Preterm PROM	0	4.7%	1.3%	0.35
FGR	6.7%	4.7%	6.6%	0.91
Placental abruption	0	0	1.3%	0.61

Patients with SCH exceeding the cutoff value (n = 29), patients with SCH below the cutoff value (n = 48), and non-SCH cases (n = 77) are shown. Patients with miscarriages were excluded from the analysis. Data are presented as mean ± SD and the ratio

*SCH* subchorionic hematoma, *SD* standard deviation, *GA* gestational age, *PROM* premature rupture of membranes, *FGR* fetal growth restriction

Moreover, p-values were assessed using the Kruskal–Wallis rank-sum test (*p < 0.05)

## Discussion

Patients who developed pregnancy complications had significantly larger SCH. The cutoff value was calculated for each method (Methods 1 and 2) in this study. A novel finding of the present study was that cases with SCH sizes exceeding the cutoff value showed a higher incidence of preterm delivery. Further, based on the AUC in the ROC analysis, Method 1 was more suitable for predicting the occurrence of pregnancy complications, although the difference was not statistically significant.

Previous studies have reported that a “larger SCH” poses a higher risk for the occurrence of pregnancy complications [6–8]. However, there has not been a defined threshold to classify the size above which an SCH is considered “large.”

In a previous study using Method 1, 46% of the patients with a ratio of ≥ 50% had experienced pregnancy-related complications [17]. In the present study, for cases with a ratio of ≥ 25% using Method 1, the rate of pregnancy complications was 31.0%, and the preterm delivery rate was 24.1% (Supplement Table 1). Therefore, we believe it is appropriate to recognize cases with a ratio of 25% or higher using Method 1 as high-risk cases.

SCHs can be accompanied by vaginal bleeding, which imposes a significant psychological and physical burden on patients. Therefore, accurate assessment of the condition and comprehensive information provision are essential. Previous reports have suggested that many cases of SCH resolve naturally and do not affect pregnancy outcomes [3, 13, 14]. In the present study, no difference in the occurrence of pregnancy complications was observed between the full SCH group (including cases with small SCHs) and the non-SCH group.

The exact pathophysiology of SCH-related adverse pregnancy outcomes has not been elucidated. Several hypotheses have been proposed regarding the mechanisms underlying the increase in perinatal complications. First, SCHs appear to be caused by immunological vasculitis in the decidual vessels [7, 18]. Immunological vasculitis may induce inflammatory cytokines, potentially increasing the risk of perinatal complications [19, 20]. Second, premature perfusion of the intervillous space before the development of placental adaptations to cope with oxidative stress may also contribute to an increase in perinatal complications. Oxidative stress may also trigger inflammation, potentially increasing the incidence of perinatal complications [21]. Third, the mechanical impact due to the presence of SCH is another potential cause. Shallow trophoblast invasion and impaired angiogenesis with resultant friable blood vessels may predispose adverse outcomes [7]. Fourth, SCH, along with an increase in intrauterine pressure, may trigger uterine contractions and potentially worsen perinatal complications.

In any of these hypotheses, a larger SCH could potentially increase perinatal complications. When SCHs are larger, there may be a greater increase in inflammatory cytokines and oxidative stress. There may be a certain threshold for an increase in perinatal complications, and the cutoff value calculated in the present study could serve as an indicator surpassing that threshold. Further investigations are necessary to determine the changes in inflammatory mediators and oxidative stress associated with hematoma size.

There are various discussions on the management of SCHs in the literature, including bed rest [22], administration of tocolytic agents, and progesterone administration [23]. However, there is no established method for improving the prognosis. In the future, it is important to identify interventions that may reduce pregnancy complications. In cases where perinatal complications occur, it is believed that by examining the aforementioned hypotheses, new insights into treatment approaches will be gained.

This study had some limitations. First, it was a retrospective study. Second, there was uncertainty regarding whether the measured cross-section of the SCH was the maximum. Furthermore, when considering perinatal outcomes, confounding factors such as bleeding and laboratory findings were not included. Finally, this study was conducted at a single institution. Further studies are required to enhance the generalizability of our findings.

## Conclusion

Large-sized SCH may indicate an increased risk of pregnancy-related complications. Moreover, recognizing and managing cases that exceed the aforementioned cutoff value as a high-risk group may be beneficial in reducing pregnancy complications.

## Supplementary Information

Below is the link to the electronic supplementary material.

Supplementary file1 (docx 13 KB)

## Data Availability

The data that support the findings of this study are available on request from the corresponding author. The data are not publicly available due to their containing information that could compromise the privacy of research participants.
