# Correction: Quantification of the size of subchorionic hematoma causing pregnancy-related complications: a retrospective cohort study

**DOI:** 10.1007/s10396-025-01540-5

**Published:** 2025-04-10

**Authors:** Tatsuya Yoshihara, Yasuhiko Okuda, Osamu Yoshino

**Affiliations:** Department of Obstetrics and Gynecology, Faculty of Medicine, University of Yamanashi, Yamanashi, Japan

**Correction: Journal of Medical Ultrasonics (2024) 51:649–654** 10.1007/s10396-024-01488-y

The article “Quantification of the size of subchorionic hematoma causing pregnancy‑related complications: a retrospective cohort study”, written by Tatsuya Yoshihara, Yasuhiko Okuda and Osamu Yoshino, was originally published under exclusive license to The Author(s), under exclusive licence to The Japan Society of Ultrasonics in Medicine. As a result of the subsequent decision to publish the article under the open access model, the article’s copyright notice was changed on 25 March 2025 to © The Author(s) 2024 and the article is now distributed under a Creative Commons Attribution 4.0 International License, which permits use, sharing, adaptation, distribution and reproduction in any medium or format, as long as you give appropriate credit to the original author(s) and the source, provide a link to the Creative Commons licence, and indicate if changes were made. The images or other third party material in this article are included in the article’s Creative Commons licence, unless indicated otherwise in a credit line to the material. If material is not included in the article’s Creative Commons licence and your intended use is not permitted by statutory regulation or exceeds the permitted use, you will need to obtain permission directly from the copyright holder. To view a copy of this licence, visit http://creativecommons.org/licenses/by/4.0

The original article has been corrected.

