# Supplementary material for: Quantification of the size of subchorionic hematoma causing pregnancy-related complications: a retrospective cohort study
Source: J Med Ultrason (2001). 2024 Aug 27;51(4):649–54. doi: 10.1007/s10396-024-01488-y (PMC11968545; doi:10.1007/s10396-024-01488-y)
Supplement: Supplementary file 1 — Supplementary file1 (docx 13 KB) [file 10396_2024_1488_MOESM1_ESM.docx]

**Supplement Table 1. The breakdown of pregnancy complications in cases with SCH occurrence**

In patients with SCH (n=80), the incidence of pregnancy-related complications and the breakdown are presented. The data are presented as a ratio (n/80). FGR was diagnosed as SGA based on the measurements obtained after birth. Placental abruption was diagnosed by an attending physician during delivery.

SCH, subchorionic hematoma; FGR, fetal growth restriction; SGA, small for gestational age; PROM, premature rupture of membranes

| Pregnancy complications | 18.9% (15/80) |
| --- | --- |
| Miscarriage | 3.8% (3/80) |
| Preterm delivery | 11.3% (9/80) |
| Preterm PROM | 3.8% (3/80) |
| FGR | 5.0% (4/80) |
| Placental abruption | 0% (0/80) |

**Supplement Table 2. Comparison of maternal background between the group with pregnancy-related complications and that without complications**

Pregnancy-related complications (n=15) and non-complications (n=65) groups are shown. Data are presented as mean ± SD and ratios. Moreover, p-values were assessed using the chi-square test, Mann–Whitney U test, and Fisher’s exact test (*p<0.05).

SD, standard deviation; ART, assisted reproductive technology; BMI, body mass index

|  | Complications  (n=15) | Non-complications  (n=65) | p-value |
| --- | --- | --- | --- |
| Age | 34.7 ± 4.1 | 34.3 ± 4.1 | 0.69 |
| Primiparous | 40.0% | 46.2% | 0.49 |
| ART | 46.7% | 46.2% | 1 |
| History of preterm delivery | 0 | 1.5% | 1 |
| Pre-pregnancy BMI | 19.7 ± 3.2 | 20.7 ± 2.7 | 0.19 |
